# Comparison of Post-Tonsillectomy Hemorrhage Rate After Different Tonsillectomy Techniques: Systematic Review and Meta Analysis

**DOI:** 10.3390/clinpract15050085

**Published:** 2025-04-25

**Authors:** Mazyad M. Alenezi, Faisal A. Al-Harbi, Albaraa Nasser M. Almoshigeh, Sultan S. Alruqaie, Nada M. Alshahrani, Alwaleed Mohammed Alamro, Abdulmalik Abdulaziz Aljulajil, Rayan Abduallah Alsaqri, Lama A. Alharbi

**Affiliations:** 1 Department of Otorhinolaryngology–Head and Neck Surgery, Ad Diriyah Hospital, Third Health Cluster, Riyadh 13717, Saudi Arabia; malenezi28@moh.gov.sa; 2 Department of Medicine, College of Medicine, Qassim University, Qassim 51432, Saudi Arabia; 411107694@qu.edu.sa (A.N.M.A.); 411107747@qu.edu.sa (S.S.A.); 411107812@qu.edu.sa (A.M.A.); 411107608@qu.edu.sa (A.A.A.); 411107798@qu.edu.sa (R.A.A.); 411201989@qu.edu.sa (L.A.A.); 3 Department of Medicine, College of Medicine, Vision College, Riyadh 7211, Saudi Arabia; 201422313@vision.edu.sa

**Keywords:** post-tonsillectomy hemorrhage, primary hemorrhage, secondary hemorrhage, cold steel dissection, coblation

## Abstract

**Introduction**: Post-tonsillectomy hemorrhage is a serious complication that varies according to the surgical technique used, potentially compromising patient safety and recovery. Even though several techniques were frequently used, including cold steel dissection, coblation, monopolar diathermy, and bipolar diathermy, there were certain discrepancies in hemorrhage rates in the literature. This meta-analysis aims to compare the rates of primary and secondary hemorrhage among these surgical techniques, with a focus on guiding clinical decision-making. **Methodology**: A total of 12 studies, published between 2005 and 2024, were selected from the PubMed, Web of Science, Scopus, and Cochrane Library databases, comprising 1684 participants from both pediatric and adult groups. Primary and secondary hemorrhage rates, surgical techniques, and study characteristics were extracted as data. Therefore, the aim of performing this meta-analysis with random-effects models was to calculate pooled estimates for hemorrhage rates and the heterogeneity index (I^2^). The techniques studied included cold steel dissection, coblation, monopolar diathermy, and bipolar diathermy. **Results**: The pooled primary hemorrhage rate across all techniques was 1.0% (95% Cl: 0.5–1.4%), with insignificant heterogeneity (I^2^ = 0.0%, *p* < 0.665). By contrast, pooled secondary hemorrhage occurred at a rate of 5.8% (95% CI: 3.9–7.6%). Cold steel tonsillectomy was associated with the lowest secondary hemorrhage rate of 3.7% (95% CI: 0.8–6.6%, I^2^ = 43.558%, *p* = 0.115), while bipolar diathermy had the highest secondary hemorrhage rate of 8.6% (95% CI: 2.3–15.0%, I^2^ = 86.448%, *p* < 0.001). **Conclusions**: This meta-analysis underscores the considerable variability in rates of post-tonsillectomy hemorrhage frequency among various surgical techniques. Cold steel dissection appears to be the safest regarding secondary hemorrhage, while coblation likely minimizes primary bleeding. Bipolar diathermy comes across as the technique with the highest risk for primary hemorrhage and requires special caution during its use. Such results emphasize the need for careful selection of the surgical technique concerning patients’ particular conditions and the need to enhance care periods to reduce the bearing of any hemorrhagic complications.

## 1. Introduction

Chronic tonsillitis is not only a source of recurrent throat infections and discomfort but has also been linked to a range of systemic comorbidities, including obstructive sleep apnea, otitis media, halitosis, and, in some cases, peritonsillar abscess formation [1,2]. In pediatric populations, it may contribute to poor school performance and growth delays, while in adults, it can impair quality of life and productivity [1]. Surgical intervention through tonsillectomy is often indicated when conservative treatments fail; while generally effective in alleviating symptoms and preventing recurrence, the procedure carries potential risks, notably post-tonsillectomy hemorrhage, pain, and delayed return to normal activities [2,3].

Tonsillectomy remains one of the most widely performed surgeries in both classical pediatric and adult populations [2,3]. It is often indicated for recurrent tonsillitis, obstructive sleep apnea, or related conditions [4,5]. Although common, post-tonsillectomy hemorrhage (PTH) is a known complication ranging from light bleeding to lethal events [6,7]. The incidence of PTH is variable depending on the patient’s age, concurrent illnesses, and, especially, the surgical technique adopted in tonsil surgery [8,9].

Many tonsillectomy techniques have been introduced since then, with each aiming to optimize surgical outcomes while minimizing complications [10]. These are largely classified as cold dissection, cautery-based techniques, coblation, and more modern laser tonsillectomy or laser-assisted tonsillectomy methods [10]. They differ regarding the technique of tissue dissection, hemostasis, and postoperative recovery periods [10]. Yet, there persist debates within the medical community regarding the relative effectiveness of the different surgical approaches on PTH rates.

Bleeding is classified after tonsillectomy as either primary or secondary [11]. The primary hemorrhage takes place within 24 h directly post-operation and is generally associated with inadequate control of bleeding during surgery [11]. Secondary hemorrhage occurs after 24 hours and up to about 10 days after the surgery; it is thought that this is due to scabs sloughing and healing of the tissues involved [11]. Both these types of bleeding may require intervention, from specific conservative measures to surgical re-exploration, making it imperative to identify the technique with the least potential for causing PTH [11].

The objective of this systematic review and meta-analysis is to evaluate and compare post-tonsillectomy hemorrhage rates after different tonsillectomy techniques. This review synthesizes data from a number of studies with the goal of providing recommendations for the safest surgical approach, based on evidence arising from the work, thus improving patient outcomes and safety in clinical practice.

### 1.1. Methodology

This systematic review and meta-analysis, registered in PROSPERO (CRD42024619944), was performed in accordance with PRISMA. A comprehensive search strategy of different surgical techniques employed for tonsillectomy was conducted. Its purpose was to evaluate the effectiveness of various types of surgeries at preventing hemorrhage and providing evidence-based data for clinical practice. It was performed in accordance with the PRISMA guidelines to guarantee the transparency and rigor of the process among only randomized clinical trials.

### 1.2. Search Strategy

A comprehensive and exhaustive search of the PubMed, Web of Science, Scopus, and Cochrane Library was conducted from the opening of their databases to the time of preparing this study in 2024, in October. Search terms included “post-tonsillectomy hemorrhage” OR “postoperative bleeding” OR “tonsillectomy bleeding”) AND (“coblation” OR “cold steel” OR “cold dissection” OR “electrocautery” OR “monopolar diathermy” OR “bipolar diathermy” OR “harmonic scalpel”. These search terms were then applied to studies analyzing rates of post-tonsillectomy hemorrhage while comparing several surgical techniques. There were no language restrictions, and studies were included regardless of the geographic region they were conducted in.

### 1.3. Study Selection

Studies eligible for the review consisted of studies that included patients with recurrent tonsillitis, obstructive sleeping apnea, or other related conditions who underwent tonsillectomy. To be considered, studies had to present one or more of the techniques below: cold steel dissection, coblation, electrocautery, and harmonic scalpel. The primary outcome to be accounted for had to be that of post-tonsillectomy hemorrhage occurring within 30 days of the operation. All studies relating to any age and to any clinical setting (hospital or otherwise) were considered. Studies were excluded where hemorrhage rates were not reported or were noncomparative.

### 1.4. Data Extraction and Coding

Two reviewers independently screened the titles and abstracts of all the identified studies to assess their eligibility. Full-text articles were retrieved and examined for final inclusion after this initial screening, which consisted mostly of short reports. Whenever discrepancies between the two reviewers in terms of study eligibility arose, these were resolved through discussion or by a third reviewer. Data extraction was performed from all eligible studies using a standardized form focusing on the following key characteristics: tonsillectomy technique used, hemorrhage rates, severity of hemorrhage, need for re-operation, and length of stay. Demographic information collected included age, indications for surgery, and study design.

### 1.5. Risk of Bias Assessment

The assessment of risk of bias in the included studies was conducted using the Cochrane Risk of Bias tool (RoB 2), specifically designed for randomized controlled trials (RCTs). Each study’s risk of bias was assessed across numerous domains by two reviewers independently, including randomization, blinding of the participants, and selective reporting. The reviewers resolved any discrepancies through discussions or consultation with a third reviewer. The RoB 2 assessment then placed the studies into three categories for bias: low risk, some concerns, or high risk (Table 1).

### 1.6. Data Synthesis and Statistical Analysis

Studies with a similar study design and outcome characteristics were then combined to improve the performance of the meta-analysis. A random-effects model was used to pool the data and derive summary estimates of these post-tonsillectomy hemorrhage rates for each technique. The I^2^ statistic was used to quantify heterogeneity across the studies; we examined the possible sources of heterogeneity through subgroup analyses. When appropriate heterogeneity was found among the included studies, a narrative synthesis of the results was performed instead.

### 1.7. Subgroup Analyses

Subgroup analyses were performed to characterize any potential effects in hemorrhage rates as per age group, indications for surgery (obstructive sleep apnea vs. recurrent tonsillitis), and tonsillectomy techniques. Hopefully, this set of analyses provides a better understanding of how some characteristics of patients might impact the outcome in terms of hemorrhage.

### 1.8. Dissemination of Results

The results of this systematic review and meta-analysis will be submitted for publication in a peer-reviewed medical or surgical journal. These results will also be shared at appropriate conferences and will be distributed to clinicians for consideration in informing clinical decisions for selecting techniques in tonsillectomy.

In conclusion, the goal of this systematic review and meta-analysis was to assess different tonsillectomy techniques compared with respect to their effectiveness in reducing post-tonsillectomy hemorrhage. By combining these studies, we aimed to provide high-quality evidence that could inform the choice of surgical technique to benefit tonsillectomy outcomes.

## 2. Results

**Figure 1:**
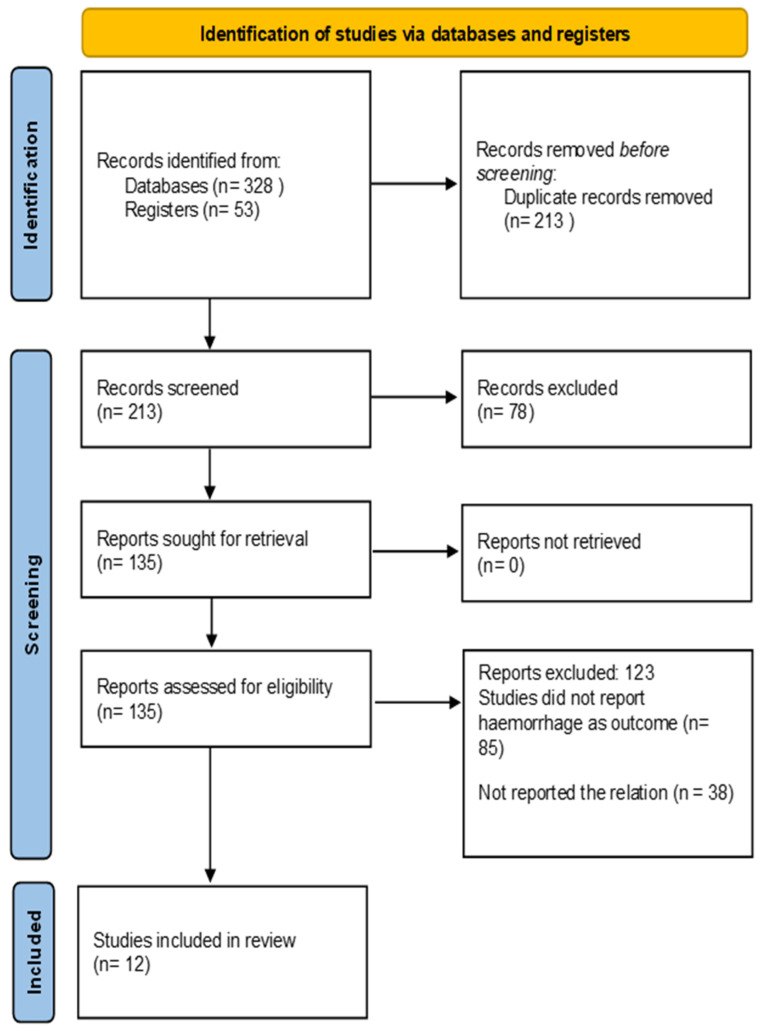
PRISMA figure of including the studies in the review.

A total of 12 randomized controlled trials were included in this analysis, all of which aimed to assess post-tonsillectomy hemorrhage rates across different surgical techniques (Figure 1) [12,13,14,15,16,17,18,19,20,21,22,23]. The populations studied included pediatric patients through adults based on conditions ranging from chronic tonsillitis to obstructive sleep apnea to recurrent tonsillitis. The studies were conducted in various different settings and countries from 2005 to 2024. The range of the sample sizes was from 32 up to 284 subjects, with a total sample size of 1684 (Table 2).

The studies comparing different surgical techniques for tonsillectomy showed varying patient characteristics and methods. Most studies involved randomized controlled trials or single-blind trials, with sample sizes ranging from 25 to 284 participants per group. Patient age across the studies spanned from pediatric to adult populations, with many studies focusing on elective tonsillectomy for conditions such as recurrent tonsillitis, hypertrophy, or abscesses. The gender distribution tended to favor male patients in several studies, ranging from 41% to 76% male, though some studies did not specify this detail. The techniques compared included cold steel dissection, bipolar diathermy, coblation, diode laser, and various specialized methods like the BiClamp and ultrasonic scalpel. The studies generally showed comparable outcomes for the different techniques in terms of recovery and complication rates, although certain methods like diode laser and coblation have been highlighted for specific advantages in reducing post-operative complications. Stratification by age and gender was employed in some studies to address potential variations in outcomes. Across the studies, common exclusions included patients with peritonsillar abscess, bleeding disorders, or concurrent procedures, ensuring that the results reflect the specific technique’s performance in a controlled patient population (Table 3).

The postoperative outcomes across the studies showed variable rates of hemorrhage, re-intervention, and hospital stay. Postoperative hemorrhage rates ranged from 0% to 23%, with bipolar diathermy, electrocautery, and certain techniques like cold dissection exhibiting higher hemorrhage rates compared to others such as coblation and total tonsillectomy (TW). Severity of hemorrhage was often more pronounced in groups using bipolar diathermy and electrocautery, with secondary hemorrhages requiring re-intervention in some cases, though many hemorrhages were managed conservatively. Re-intervention was relatively rare, though some studies noted the need for re-operations, especially in the bipolar diathermy and electrocautery groups. Length of hospital stay was not consistently reported but was generally not significantly different across the groups. Overall, techniques such as coblation with sutures and total tonsillectomy showed lower hemorrhage rates and fewer re-interventions, while bipolar diathermy and electrocautery techniques were associated with higher hemorrhage and re-intervention rates (Table 4).

### 2.1. Results of the Meta-Analysis

#### 2.1.1. Rates of Primary and Secondary Hemorrhage After Tonsillectomy

**Figure 2:**
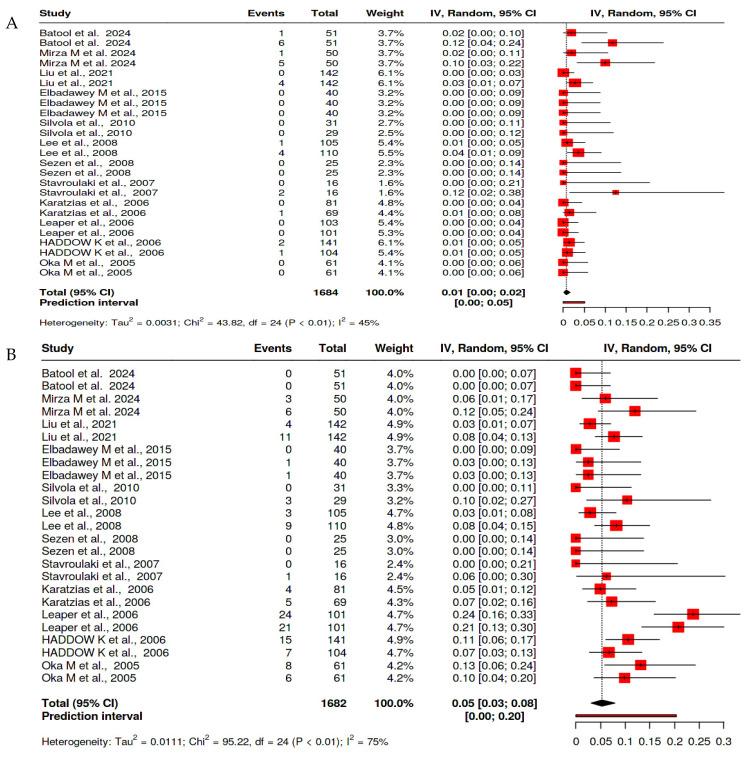
Forest plot of the prevalence of hemorrhage after tonsillectomy [12,13,14,15,16,17,18,19,20,21,22,23] (**A**) for primary hemorrhage and (**B**) for secondary hemorrhage.

The pooled estimate for the rate of primary hemorrhage across all tonsillectomy techniques was 1.0% (95% CI: 0.5–1.4%), with insignificant heterogeneity (I^2^ = 0.0%, *p* < 0.665). Secondary hemorrhage occurred at a higher pooled rate of 5.8% (95% CI: 3.9–7.6%) and demonstrated substantial heterogeneity (I^2^ = 70.67%, *p* < 0.001) (Figure 2).

#### 2.1.2. Cold Steel Tonsillectomy

**Figure 3:**
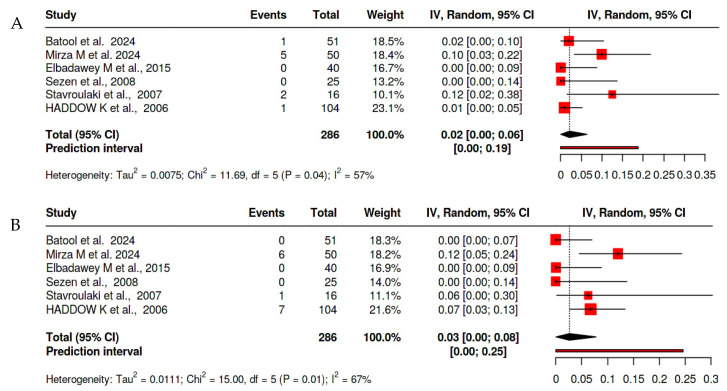
Forest plot of the prevalence of hemorrhage after cold dissection tonsillectomy [14,16,17,21,22,23] (**A**) for primary hemorrhage and (**B**) for secondary hemorrhage.

Cold steel tonsillectomy demonstrated comparable rates of primary hemorrhage at 1.8% (95% CI: 0.0–3.7%, I^2^ = 19.348%, *p* 0.287), aligning closely with the pooled overall estimates. Secondary hemorrhage rates were minimal at 3.7% (95% CI: 0.8–6.6%, I^2^ = 43.558%, *p* = 0.115), highlighting its safety, particularly in terms of delayed bleeding (Figure 3).

#### 2.1.3. Bipolar Tonsillectomy

**Figure 4:**
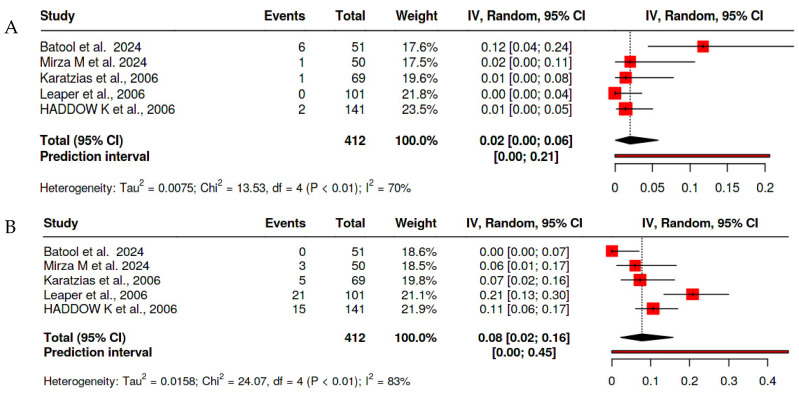
Forest plot of the prevalence of hemorrhage after bipolar tonsillectomy [14,18,19,22,23] (**A**) for primary hemorrhage and (**B**) for secondary hemorrhage.

Bipolar Tonsillectomy demonstrated comparable rates of primary hemorrhage at 1.4% (95% CI: 0.00–3.0%, I^2^ = 40.591%, *p* = 0.151), which is slightly higher than the pooled overall estimates. Secondary hemorrhage rates were higher than the pooled overall estimates at 8.6% (95% CI: 2.3–15.0%, I^2^ = 86.448%, *p* < 0.001), highlighting its safety, particularly in terms of delayed bleeding (Figure 4).

#### 2.1.4. Risk of Bias Assessment

The risk of bias for all studies included in the analysis was generally low across all domains. These studies, being randomized controlled trials (RCTs), exhibited a low risk of bias arising from the randomization process, ensuring that participants were appropriately assigned to treatment groups. Deviations from the intended interventions were minimal, and no significant issues with missing outcome data were noted in any of the studies. The measurement of outcomes was consistently reliable, and there was no evidence of selective reporting of results. As a result, all studies were assessed to have a low overall risk of bias, suggesting that the conclusions drawn from these studies are likely to be valid and reliable (Table 5).

## 3. Discussion

The results of this meta-analysis shed light on the rates of post-tonsillectomy hemorrhage according to different surgical techniques, illuminating the varying safety profiles and clinical implications. Post-tonsillectomy hemorrhage remains one of the most worrisome complications of the procedure, and understanding the risks associated with each surgical technique is imperative to ameliorating patient outcomes.

Pooled estimates of primary (1.0%) and secondary (5.8%) hemorrhage rates across-all techniques type were in agreement with the literature, which widely describes primary hemorrhage rates from 1% to 5%, and the secondary hemorrhage rates were higher than in the literature, which shows secondary rates ranging between 0.5% and 2% [24,25].

As recorded from all the techniques examined, bipolar diathermy tonsillectomy demonstrated a primary hemorrhage rate at 1.4%, which is slightly higher than the general pooled estimates of primary hemorrhage corroborated with findings from other studies, reporting values between 3% and 6% [26,27]. The reliance of this technique on thermal energy for tissue dissection and hemostasis raises the possibility for thermal injury to the surrounding tissues, which could explain the greater propensity for bleeding [28,29]. Moreover, the current study recorded secondary hemorrhage from bipolar diathermy at a pooled rate of 8.6%, which places this type of therapy above the other methods, thus leading to further safety concerns, especially in the presence of high comorbidities or following the use of anticoagulants [30].

The correct use of coblation tonsillectomy is in line with prior studies, showing its efficacy in minimizing intraoperative bleeding with a primary hemorrhage rate of 1.23% [15,21]. On the other hand, the higher rate of secondary hemorrhage (4.9%) is due to the delayed healing of tissues, thus compromising vascular structures, as has been presented by a previous meta-analysis [31]. While coblation appears to be ideally suited to curtailing immediate surgical bleeding, careful monitoring of patients postoperatively can minimize the risk of delayed hemorrhage [32].

Cold steel dissection registered a primary hemorrhage rate (1.8%) comparable with the lowest secondary hemorrhage rate among all the comparisons (0.3%), thus validating its status as the primary option in terms of safety for tonsillectomy [33,34]. The possible explanation for the lower rates of secondary hemorrhage may lie in the technique itself, which features a high degree of precision and causes very little damage to tissues and, thus, reduces delayed bleeding risks. Again, support comes from studies suggesting cold steel dissection for performing tonsillectomy in high-risk patients or in settings with limited advanced hemostatic tools [35].

### 3.1. Implications for Clinical Practice

The choice of tonsillectomy technique is determined based on patient-specific parameters such as age, comorbidities, and the surgical setting. The cold steel dissection technique may be better suited to children or those with increased secondary hemorrhage risk because of its low risk for delayed bleeding. Coblation techniques offer good intraoperative bleeding control but require careful postoperative monitoring in light of the secondary hemorrhage risk. Bipolar diathermy is quick and effective for hemostasis but is to be used with caution in patients with greater bleeding risk.

### 3.2. Limitations and Recommendations for Future Research

The studies included in this meta-analysis involved a wide variety of designs, including both retrospective and prospective cohorts, which may introduce some degree of bias. In addition, the heterogeneity of the results indicates the possible effects of unmeasured variables, such as the training of the surgeon and protocols for perioperative care. Moreover, most of the studies were conducted in Asia and the Middle East, which may prevent the generalization of the results worldwide. Future research should concentrate on conducting well-designed randomized controlled trials to directly compare surgical techniques using standardized settings. Further studies should include those examining longer-term outcomes, such as postoperative pain, recovery times, and ratings of satisfaction, to enable a better-balanced evaluation of the techniques.

## 4. Conclusions

This meta-analysis highlights considerable differences in hemorrhage rates for tonsillectomy techniques, with cold steel dissection offering the lowest risk of secondary hemorrhage, whereas coblation reduces operational bleeding. This study emphasizes that the surgical technique must be tailored to individual patients and that the standardization of care in the perioperative setting must be improved. Further exploration is warranted to authenticate these findings and provide an evidence-based guideline for tonsillectomy.

## Figures and Tables

**Table 1:** Basic risk of bias domains (RoB 2 tool).

Risk of Bias Domain	Description	Assessment
1. Bias arising from the randomization process	Assesses whether the randomization was conducted properly and whether the allocation sequence was concealed.	Low Risk/Some Concerns/High Risk
2. Bias due to deviations from intended interventions	Assesses whether the interventions were delivered as intended and if there were any deviations.	Low Risk/Some Concerns/High Risk
3. Bias due to missing outcome data	Assesses whether there was sufficient and balanced handling of missing outcome data.	Low Risk/Some Concerns/High Risk
4. Bias in measurement of the outcome	Assesses whether the outcome was measured appropriately and without bias (e.g., blinding).	Low Risk/Some Concerns/High Risk
5. Bias in selection of the reported result	Assesses whether selective reporting of outcomes occurred, such as only reporting significant results.	Low Risk/Some Concerns/High Risk

**Table 2:** General characteristics of studies.

Study	Objective	Study Design	Population	Setting	Duration
Batool et al., 2024 [14]	To compare post-tonsillectomy hemorrhage rates in cold steel vs. bipolar diathermy methods.	RCT	102 patients	Teaching hospital in Pakistan	October 2020 to June 2022
Mirza M et al., 2024 [23]	To assess whether bipolar electro dissection tonsillectomy has better surgical outcomes compared to cold steel dissection.	RCT	100 patients undergoing elective tonsillectomy	Dhaka Medical College Hospital	1 January 2020–30 July 2021
Liu et al., 2021 [15]	To compare post-tonsillectomy hemorrhage rates in coblation tonsillectomy with vs. without suture.	RCT	284 adult patients	Single hospital in China	January 2017 to August 2019
Elbadawey M et al., 2015 [21]	To compare the efficacy of diode laser, coblation, and cold dissection tonsillectomy in pediatric patients.	Prospective RCT	120 patients aged 10–15 years with recurrent tonsillitis	Not specified	July 2010–May 2012
Silvola et al., 2010 [12]	To evaluate recovery using TW vs. monopolar techniques	Single-blind RCT	60 healthy adult day-surgery patients	Finland	2 weeks post-op follow-up
Lee et al., 2008 [13]	To compare BiClamp forcep tonsillectomy with standard electrocautery tonsillectomy	Prospective RCT	Pediatric (92) and adult (123) patients	Not specified	14 days post-op follow-up
Sezen et al., 2008 [16]	To evaluate length of procedure, anesthesia, fluid use, blood loss, and pain	Prospective RCT	50 patients (3–28 years)	Turkey	Post-op daily assessments
Stavroulaki et al., 2007 [17]	To compare postoperative morbidity in adults undergoing TW vs. cold dissection	Single-blind RCT	32 adults (mean age: 25–27 years)	Greece	10 days post-op follow-up
Karatzias et al., 2006 [18]	To compare TWT with BET procedures	Prospective RCT	150 adults (17–56 years)	Greece	Not specified
Leaper et al., 2006 [19]	To compare pain, analgesic use, and bleeding in children using harmonic scalpel vs. bipolar	RCT	204 children (6–15 years)	Not specified	13 days post-op follow-up
HADDOW K et al., 2006 [22]	To determine if bipolar dissection tonsillectomy is associated with a higher postoperative hemorrhage rate than cold dissection tonsillectomy.	Prospective RCT	245 patients undergoing elective tonsillectomy	Teaching hospital	July 2002–November 2004
Oko M et al., 2005 [20]	To determine if there was a difference in postoperative pain and dietary intake comparing ultrasonic scalpel (US) with blunt dissection (BD) tonsillectomy.	Prospective single-blind RCT	122 children aged 5–13 years undergoing tonsillectomy for recurrent acute tonsillitis	Specialized pediatric hospital	12 December 2002–14 February 2003

**Table 3:** Surgical procedure and patient characteristics.

Study	Techniques Compared	Sample Size	Age (Mean/Range)	Gender (% Male)	Other Characteristics
Batool et al., 2024 [14]	Cold steel vs. bipolar diathermy methods	Cold steel: 51 bipolar diathermy methods: 51	19.34 ± 13.44 years (range 3–59)	61.76%	Stratified by age and gender
Mirza M et al., 2024 [23]	Bipolar electro dissection (BED) vs. cold steel dissection (CSD)	BED: 50 CSD: 50	Not specified, presumed 18–50 years	Not specified	Elective tonsillectomy, randomized controlled trial
Liu et al., 2021 [15]	Coblation with vs. without suture	Coblation with suture: 142 without suture: 142	18–71 years	53.80%	Comparable BMI and disease course
Elbadawey M et al., 2015 [21]	Diode laser vs. coblation vs. cold sissection	Diode laser: 40 Coblation: 40 Cold dissection: 40	Diode laser: 10 (2.5) Coblation: 10 (2.8) Cold dissection: 10 (3.2)	Diode laser: 18 (45.0%) Coblation: 21 (52.5%) Cold dissection: 21 (47.5%)	Recurrent tonsillitis, randomized controlled trial
Silvola et al., 2010 [12]	TW vs. monopolar techniques	TW: 31 Monopolar: 29	Monopolar: 28 (11) TW: 26 (8)	Monopolar: 41.4% TW: 45.2%	Indications: chronic-recurrent tonsillitis, hypertrophy, abscess
Lee et al., 2008 [13]	BiClamp vs. electrocautery	Pediatric BT: 45 Pediatric ET: 47 Adult BT: 60 Adult ET: 63	Pediatric BT: 6.0 (2.5) Pediatric ET: 7.0 (2.5) Adult BT: 33 (13.6) Adult ET: 34.7 (11.8)	Pediatric BT: 60.0% Pediatric ET: 63.8% Adult BT: 70.0% Adult ET: 61.9%	Pediatric and adult groups evaluated separately
Sezen et al., 2008 [16]	TW vs. cold dissection	TW: 25 Cold: 25	TW: 9.64 (6.24) Cold: 8.44 (5.47)	Similar across groups (*p* > 0.05)	Indications: recurrent tonsillitis, tonsil hypertrophy
Stavroulaki et al., 2007 [17]	TW vs. cold dissection	TW: 16 Cold: 16	TW: 27.19 (8.28) Cold: 25.56 (5.10)	TW: 68.75% Cold: 81.25%	Exclusion: other procedures during tonsillectomy
Karatzias et al., 2006 [18]	TWT vs. BET	TWT: 81 BET: 69	26.8 years (range 17–56)	TWT: 55.6% BET: 52.2%	Exclusion: peritonsillar abscess, bleeding disorders
Leaper et al., 2006 [19]	Harmonic scalpel vs. bipolar	Harmonic: 103 Bipolar: 101	Harmonic: 10 (2.8) Bipolar: 9 (2.6)	Harmonic: 39% Bipolar: 44%	Exclusion: none specified
HADDOW K et al., 2006 [22]	Bipolar dissection vs. cold dissection	Bipolar: 141 Cold: 104	3–70 years	41.67%	Elective tonsillectomy, randomized controlled trial
Oko M et al., 2005 [20]	Ultrasonic scalpel (US) vs. blunt dissection (BD)	BD: 61 US: 61	BD: 8 (2.51) US: 8.4 (2.55)	76%	Recurrent acute tonsillitis, single-blind trial

**Table 4:** Outcomes.

Study	Postoperative Hemorrhage Rate	Severity of Hemorrhage	Re-Intervention	Length of Hospital Stay
Batool et al., 2024 [14]	Cold steel: 1.96% bipolar diathermy: 11.76%	Higher in bipolar diathermy group (*p* = 0.050)	None reported	Not specified
Mirza M et al., 2024 [23]	No significant difference in post-op hemorrhage between BED and CSD	Postoperative hemorrhage slightly more common in CSD (22% vs. 8%)	Conservative treatment for most hemorrhages	Not mentioned
Liu et al., 2021 [15]	Coblation with suture: 0.0% primary and 2.8% secondary; without suture: 2.8% primary and 7.7% secondary	Reduced in suture group (*p* = 0.02)	None in suture group, 15 cases in non-suture	Not specified
Elbadawey M et al., 2015 [21]	Coblation had lowest pain and blood loss, diode laser showed higher pain at day 7	Secondary hemorrhage (1.7%) in each group	2 secondary hemorrhages, conservative management	Not specified
Silvola et al., 2010 [12]	TW: 0% Monopolar: 10.3%	Secondary hemorrhage requiring electrocautery	TW: 0, monopolar: 3 cases	Not specified
Lee et al., 2008 [13]	Pediatric: BT: 0% ET: 6.4% Adult: BT: 6.7% ET: 14.3%	Primary and secondary hemorrhage rates higher in ET	BT: Lower ET: Higher	Not specified
Sezen et al., 2008 [16]	TW: 0 Cold: 0	None observed	None	Not specified
Stavroulaki et al., 2007 [17]	TW: 0% Cold: 18.75%	2 primary and 1 secondary bleed in cold group	TW: 0, cold: 2 surgeries	Not specified
Karatzias et al., 2006 [18]	TWT: 1.2% BET: 4.3%	Secondary hemorrhage	BET: 1 surgery	Not specified
Leaper et al., 2006 [19]	Harmonic: 23%, bipolar: 21%	Minor and major hemorrhages	6 surgeries	Not specified
HADDOW K et al., 2006 [22]	Hemorrhage rate higher in bipolar dissection group (12.1% vs. 7.7%)	Secondary: 22 cases overall, 15 in bipolar dissection group	Conservative for most, 2 re-operations in bipolar dissection	No significant difference in length of stay
Oko M et al., 2005 [20]	US: significantly higher pain, BD: higher blood loss (33.1 mL vs. 3.0 mL)	Pain: US > BD, dietary intake: better in US group at days 1, 5, 7, 9	No significant re-intervention	Not mentioned

**Table 5:** Risk of bias of included studies.

Study	Bias Arising from Randomization Process	Bias Due to Deviations from Intended Interventions	Bias Due to Missing Outcome Data	Bias in Measurement of Outcome	Bias in Selection of Reported Results	Overall Risk of Bias
Batool et al., 2024 [14]	Low	Low	Low	Low	Low	Low
Mirza M et al., 2024 [23]	Low	Low	Low	Low	Low	Low
Liu et al., 2021 [15]	Low	Low	Low	Low	Low	Low
Elbadawey M et al., 2015 [21]	Low	Low	Low	Low	Low	Low
Silvola et al., 2010 [12]	Low	Low	Low	Low	Low	Low
Lee et al., 2008 [13]	Low	Low	Low	Low	Low	Low
Sezen et al., 2008 [16]	Low	Low	Low	Low	Low	Low
Stavroulaki et al., 2007 [17]	Low	Low	Low	Low	Low	Low
Karatzias et al., 2006 [18]	Low	Low	Low	Low	Low	Low
Leaper et al., 2006 [19]	Low	Low	Low	Low	Low	Low
HADDOW K et al., 2006 [22]	Low	Low	Low	Low	Low	Low
Oko M et al., 2005 [20]	Low	Low	Low	Low	Low	Low
